# Assessment of sarcopenia tools as predictors of falls in patients with mild to moderate Parkinson's Disease: A cohort study

**DOI:** 10.1016/j.clinsp.2025.100776

**Published:** 2025-09-18

**Authors:** Danielle Pessoa Lima, Vlademir Carneiro Gomes, João Rafael Gomes de Luna, Lucas Tadeu Rocha Santos, Samuel Brito de Almeida, Antonio Brazil Viana-Júnior, Carlos Eduardo Urbano da Silva, Thais de Menezes Dantas, Carla Marineli Saraiva do Amaral, Arnaldo Aires Peixoto Junior, Jarbas de Sá Roriz-Filho, Renan Magalhaes Montenegro-Júnior, Pedro Braga-Neto

**Affiliations:** a Division of Geriatrics, Department of Clinical Medicine, Universidade Federal do Ceará, Fortaleza, CE, Brazil; b Medical School of Universidade de Fortaleza, Fortaleza, CE, Brazil; c Clinical Research Unit of Hospital Universitário Walter Cantídio, Universidade Federal do Ceará/Empresa Brasileira de Serviços Hospitalares (EBSERH), Fortaleza, CE, Brazil; d Graduate program in Physical Education, Universidade Federal do Ceará, Fortaleza, CE, Brazil; e Division of Neurology, Department of Clinical Medicine, Universidade Federal do Ceará, Fortaleza, CE, Brazil; f Center of Health Sciences, Universidade Estadual do Ceará, Fortaleza, CE, Brazil

**Keywords:** Falls, Recurrent falls, Sarcopenia, Parkinson’s Disease, Sarcopenia Screening Tools

## Abstract

**Background:**

Sarcopenia contributes significantly to falls, frailty, and functional decline. Falls result from multiple factors.

**Objectives:**

To assess whether sarcopenia assessment tools can predict falls and recurrent falls in patients with mild to moderate Parkinson’s Disease (PD).

**Methods:**

The authors conducted a cohort study from March 2021 to March 2023 at a Brazilian tertiary public hospital, including patients aged 50 or older with mild to moderate PD who could stand and walk independently. The authors utilized tests recommended by the revised European consensus on sarcopenia (2019): SARC-F, grip strength, Short Physical Performance Battery, and body composition via DEXA scan. SARC-F is a cost-effective tool for assessing sarcopenia risk, evaluating perceived physical limitations. Participants completed a standardized fall report and were monitored monthly for twelve months.

**Results:**

Among 103 patients, 48 (46.6 %) experienced falls, and 23 (22.3 %) had recurrent falls, totaling 159 falls. Longer disease duration predicted both falls and recurrent falls over 12-months. A history of falls in the last six months and dysautonomia also predicted falls, while SARC-F scores and type-2 diabetes predicted recurrent falls. SARC-F accuracy ranged from 58.06 % at a score of 4 % to 78.64 % at 10, with a correlation to UPDRS Part III.

**Conclusions:**

SARC-F, disease duration, and type-2 diabetes were independent predictors of recurrent falls. A history of falls, disease duration, and dysautonomia predicted falls. Confirmed sarcopenia did not predict falls or recurrent falls in this cohort.

## Highlights

• Predictors of falls were assessed in patients with mild to moderate Parkinson’s disease.
• Higher SARC-F scores and type-2 diabetes independently predicted recurrent falls.
• Longer disease duration and dysautonomia were independent predictors of falls.
• Confirmed sarcopenia did not predict falls in the 12-month cohort study.
• Future studies should include specific tools for sarcopenia assessment in Parkinson’s disease.

## Introduction

Sarcopenia is an important determinant of falls, frailty and functional decline.^1^ It is defined as a muscular disease marked by reduced muscle mass, strength and function.^1^ Recent changes have occurred in the concept of sarcopenia due to the introduction of strength and function assessment, as well as the recognition of the disease in ICD-10 in 2016.^2^

Muscle mass declines annually by 1 % to 2 % starting from the age of 50, while muscle strength decreases by approximately 1.5 % annually between the ages of 50 and 60, accelerating to a rate of 3 % per year thereafter.^3^ According to a recent review, the estimated prevalence of sarcopenia in the general population is about 10 % to 16 % of the elderly worldwide.^4^

There is evidence pointing to a higher prevalence of sarcopenia in patients with chronic and neurodegenerative conditions like Parkinson's Disease (PD); however, the studies used different diagnostic methods and consensus criteria.^5^^,^^6^ Moreover, the sit-to-stand test is not appropriate for assessing strength in PD.^7^ Sarcopenia and PD have shared pathogenesis of inflammation, oxidative stress, malnutrition and reduced physical activity.^8^

Barichella and colleagues showed lower prevalences of sarcopenia and dynapenia in patients with PD in an Italian cohort compared to other Parkinsonian syndromes and age-matched controls, although there was a correlation with the duration and severity of the disease. Rigidity and tremor, as well as treatment with levodopa ‒ which potentially induces Growth Hormone (GH) secretion and dyskinesia ‒ may influence the less affected body composition in PD, pointing for the need of more studies.^9^

Falls are a frequent complaint among PD patients. Single and recurrent fallers may differ according to various functional characteristics.^10^ There is scarce information about the similarities and differences across these groups.^11^ Falls are secondary to multifactorial factors, but there is no consensus that the recommended tests by the Revised European Consensus of Sarcopenia (EWGSOP2)^1^ are appropriate for PD.^1^

Based on these findings, the aim of this study was to evaluate if the assessment tools for sarcopenia, such as the SARCF test, appendicular lean mass, grip strength, and physical performance, are predictors of falls and recurrent falls in patients with mild to moderate PD.

## Methods

### Study participants

The authors performed a cohort study between March 2021 to March 2023 at a Brazilian public tertiary hospital. The sample consisted of subjects with PD regularly followed at the Movement Disorders outpatient clinic of Hospital Universitário Walter Cantídio (HUWC). The diagnosis of PD was confirmed by two neurologists and one geriatrician specialized in PD using the Movement Disorders Society (MDS) criteria. Patients were recruited according to the following criteria: confirmed clinical diagnosis of PD, disease severity score of 1 to 3 on the modified Hoehn and Yahr scale (HY), and aged at least 50 years old. Subjects with severe medical issues or uncontrolled chronic disorders that could interfere with the clinical assessment were excluded from the study, or conditions that could make the Dual Energy X-Ray Absorptiometry (DEXA) interpretation difficult (recent gastrointestinal contrast or radionuclide administration within the last 72 hours; Deep Brain Stimulation; Heart Pacemaker).

The HUWC Research Ethics Committee approved the study since all subjects provided written informed consent (register n° 91075318.1.0000.5045). The study's researchers interviewed and evaluated each patient.

### Clinical assessment

The authors used a structured interview to collect sociodemographic and medical information. The authors evaluated prior histories of clinical conditions. The clinical data collected from the patients were compared with data from their family members, caregivers, and medical records. The authors additionally gathered information on the antiparkinsonian drugs used and evaluated Activities of Daily Living (ADL) using the Schwab and England ADL (SE ADL) Scale, PD severity using the HY staging, and motor parkinsonian symptom severity using the Movement Disorders Society-Unified Parkinson's Disease Rating Scale part III (UPDRS-III). The Postural Instability Gait Disorder (PIGD) score was calculated by combining 4-items from the UPDRS Part III: 3.9 sit-stand, 3.10 gait, 3.12 postural stability and 3.13 posture. Lower Limb Bradykinesia (LLB) was calculated by combining 3-items from the UPDRS-III: 3.7 toe tapping, 3.8 leg agility, and 3.9 arising from chair. The 15-item Geriatric Depression Scale (GDS-15) was used to measure depressive symptoms, while the Mini-Mental Status Examination (MMSE) was used to test cognitive performance. All participants were weighed without shoes on or any heavy accessories such as mobile phones and wallets. The body mass index was determined by dividing the total body weight (in kilograms) by the square of the height (meters).

### Sarcopenia assessment

Low handgrip strength^1^ was used as a sign of probable sarcopenia. According to the EWGSOP2, confirmed sarcopenia was diagnosed as poor muscle strength and low muscle quantity or quality, with low muscle strength, low muscle amount or quality, and low physical performance being considered severe.

All patients answered the SARC-F test. The SARC-F sarcopenia risk assessment method is a straightforward and cost-free method in community healthcare settings and other clinical settings.^1^ The first question evaluates muscle strength (how much difficulty do you have in lifting and carrying 10 pounds?); the second question evaluates assistance in walking (how much difficulty do you have walking across a room?); the third question evaluates their ability to get up from a chair (how much difficulty do you have transferring from a chair or bed?); the fourth question evaluates their ability to climb stairs (how much difficulty do you have climbing a flight of 10 stairs); and the fifth question evaluates the frequency of falls (how many times have you fallen in the past year?). Every item has a score range of 0 to 2, where 0 represents no difficulty, 1 represents moderate difficulty, and 2 represents extreme difficulty or incapacity to do the task. Regarding the question of falls frequency, 0 means they have never fallen in the past year; 1 means they have fallen one to three times; and 2 means they have fallen four or more times.

The EWGSOP2′s guidelines were used to evaluate handgrip strength measurement and cut-offs (27 kg for men and 16 kg for women). A SAEHAN® dynamometer was used in accordance with the Southampton protocol (three trials on each side, alternating sides, and the maximal grip score from all six trials used).

Physical performance was assessed using the Short Physical Performance Battery (SPPB) exam.^1^ The exam includes measurements of standing balance, 4 m gait speed, and the time needed to get up from a chair five times. The patients were told to maintain their balance by standing with their feet together before spending 10 seconds in each of the semi-tandem and tandem positions, which involve positioning one heel near the other foot's big toe. Next, the participants were asked to walk down an 8 m track (consisting of 2 m of acceleration and 2 m of deceleration) at their usual rate to assess their gait speed using a stopwatch. Then, they were directed to stand up and sit down as quickly as they could five times while keeping their arms crossed over their chests to evaluate their ability to get up from a chair. This was not done until after the subject had demonstrated that he/she could stand up unaided in one motion. The total SPPB score was subsequently calculated. The greatest score is 12, and a score of less than 8 points indicates inadequate physical performance.

Lean mass index (LMI = ASMM/Ht2) was calculated by estimating Appendicular Skeletal Muscle Mass (ASMM) using DEXA and adjusting for height in meters squared. Appendicular lean mass was measured as lean mass in the arms and legs. Appendicular lean mass measures were generated by multiplying the values for the unaffected side by two for individuals whose body parts had unilateral damage. According to the EWGSOP2, low muscle mass was defined as an ASMM index of less than 7 kg/m^2^ for men and less than 5.5 kg/m^2^ for women.^1^ All patients underwent evaluations for illness staging, UPDRS-III, GDS-15, MMSE, SPPB, and handgrip strength during the medication “on” stages. An inelastic tape measure was used to measure the right Calf Circumference (CC) at the right calf's largest girth.

### Falls assessment

A fall was defined as the patient falling unintentionally to the ground or to a lower level, which was not due to a seizure, a car accident, a bicycle accident, or syncope. Patients were questioned about any similar occurrences in the previous one to six months prior to the survey. Data on falls were verified with data from families, caregivers, and clinical records to ensure accuracy. Patients were recruited during the consultation. Those who agreed to participate in the study underwent physical tests and received a physical questionnaire to fill out at home about the circumstances of the falls. Furthermore, they received monthly telephone calls during the 12-month period to investigate prospective falls.

### Statistical analysis

The study data were collected and managed using the electronic data collection and management tool REDCap, hosted at the Clinical Research Unit of the same University Hospital.

The participants were categorized as non-fallers × fallers (1 fall or more), and non-recurrent-fallers × recurrent fallers (2 falls or more) after 12- months of follow-up.

The variables were presented as mean, standard deviation and median, frequency and prevalence rate. The Mann-Whitney *U* test and Student’s *t*-test were used in the analysis of the clinical and demographic variables of the participants, verifying the adherence of the data to the Gaussian distribution. Pearson's Chi-Squared test and Fisher's exact test were used to investigate the association between categorical variables.

The sample power was 99 % and calculated, a posteriori, using the G Power 3.1.2 software based on the sarcopenia and falls variables.

Logistic regression was performed to assess variables independently associated with the outcome of recurrent falls, considering those that exhibited statistically significant associations in the bivariate analysis. The logistic regression model was used to generate a Receiver Operating Characteristic (ROC) analysis. A significance level of 5 % was adopted. Statistical analyses were performed using the R and Microsoft Excel 2016 statistical programs.

## Results

**Fig. 1:**
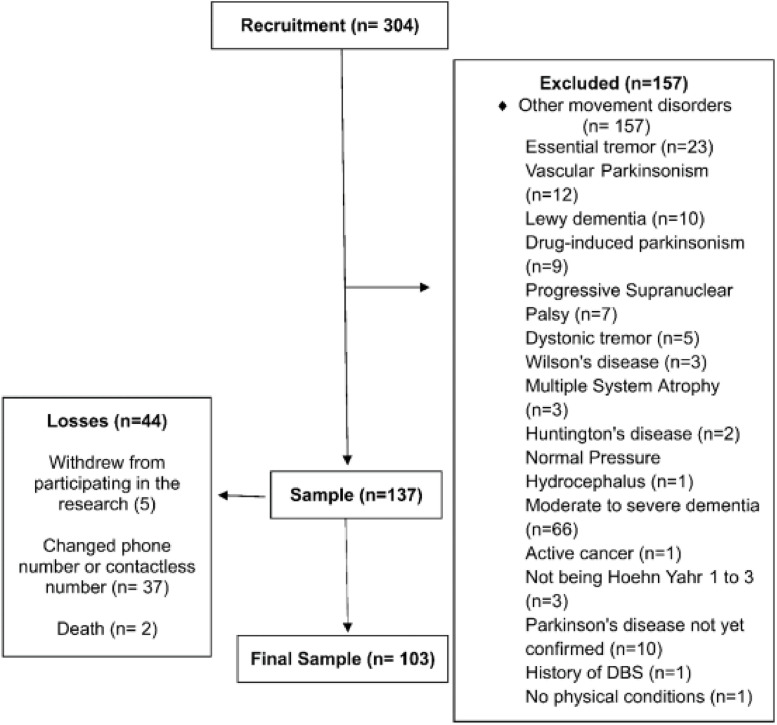
Study flow chart.

The sample for the present study consisted of 103 patients (Fig. 1), among whom 48 (46.6 %) were classified as fallers and 23 (22.3 %) as recurrent fallers. The mean age was 66 ± 11 years, and the mean disease duration was 9.9 ± 6 years, with 38 (36.9 %) of the patients being female. The most prevalent comorbidities were Hypertension (*n* = 46, 45 %), Dyslipidemia (*n* = 14, 14 %) and type-2 Diabetes (*n* = 10, 9.7 %) (Supplementary Table). A total of 30 patients (29 %) were diagnosed as having depression in the evaluation of the depression criteria by the Diagnosis and Statistical Manual of Mental Disorders, Fifth Edition (DSM-V). A total of 159 falls were reported. The mean levodopa dosage of the sample was 762 ± 328 mg, and the mean UPDRS-III was 40 ± 13. Moreover, 36 patients (35 %) had at least one fall in the previous 6-months.

Table 1 presents the bivariate analysis of the key variables for the study groups. Bivariate analyses of all the collected clinical and demographic variables with the outcomes of falls and recurrent falls are available in the Supplementary Material. The following factors showed statistically significant correlations with incidental falls: lower SE ADL scores, longer disease duration, more concurrent medications, worse MMSE scores, higher PIGD and LLB scores, reduced gait speed, higher PIGD and LLB scores, decreased physical performance based *On SPPB* scores, need for a walking aid, alcohol use, not exercising for at least 30 minutes twice a week, dysautonomia, visual hallucinations, use of antidepressants, antihypertensives, benzodiazepines, and anticholinesterase inhibitors, and a more advanced stage of PD according to HY staging. Depression, higher scores on the SARC-F, longer disease duration, a history of falls within the last 6 months, and elevated scores on the GDS were associated with both incidental and recurrent falls. Having dysautonomia and type-2 Diabetes was associated with recurrent falls. Table 1 and Supplementary Table present these results.

**Table 1:** Bivariate analysis of clinical and demographical variables with falls and recurrent falls*.*

Variables	0 fall (*n* = 551)	≥1 fall (*n* = 481)	p	0‒1 fall (*n* = 801)	**≥ 2 fall** **(*n* = 231)**	**p**
**Clinical aspects**						
Gender			0.348			0.458
Female	18 (33 %)	20 (42 %)		28 (35 %)	10 (43 %)	
Male	37 (67 %)	28 (58 %)		52 (65 %)	13 (57 %)	
Age (years)	66 ± 10 (63)	66 ± 11 (69)	0.997	66 ± 11 (69)	65 ± 10 (65)	0.716
Weight (kg)	67 ± 16 (66)	66 ± 13 (66)	0.830	66 ± 15 (66)	68 ± 14 (64)	0.502
Height (m)	1.60 ± 0.09 (1.60)	1.58 ± 0.09 (1.59)	0.306	1.59 ± 0.09 (1.59)	1.59 ± 0.10 (1.60)	0.902
Hypertension	28 (51 %)	18 (38 %)	0.172	37 (46 %)	9 (39 %)	0.545
Type-2 Diabetes	4 (7.3 %)	6 (13 %)	0.508	5 (6.3 %)	5 (22 %)	0.042
Use of walking aid	6 (11 %)	16 (33 %)	**0.006**	14 (18 %)	8 (35 %)	0.088
Physical active^a^	32 (58 %)	15 (31 %)	**0.006**	37 (46 %)	10 (43 %)	0.814
Depression	10 (18 %)	20 (42 %)	**0.009**	19 (24 %)	11 (48 %)	0.025
Number of medicines	4.83 ± 2.13 (5.00)	5.75 ± 2.24 (5.50)	**0.039**	5.10 ± 2.11 (5.00)	5.74 ± 2.56 (5.00)	0.372
Antidepressives	17 (31 %)	24 (50 %)	**0.048**	28 (35 %)	13 (57 %)	0.063
Antihypertensive	23 (42 %)	11 (23 %)	**0.042**	30 (38 %)	4 (17 %)	0.071
Benzodiazepine	2 (3.6 %)	9 (19 %)	**0.013**	6 (7.5 %)	5 (22 %)	0.065
Atypical antipsychotic	2 (3.6 %)	2 (4.2 %)	>0.999	3 (3.8 %)	1 (4.3 %)	>0.999
Cholinesterase inhibitor	0 (0 %)	5 (10 %)	0.020	3 (3.8 %)	2 (8.7 %)	0.310
GDS	4.11 ± 2.95 (4.00)	6.00 ± 3.52 (5.50)	0.005	4.60 ± 3.11 (4.00)	6.39 ± 3.83 (6.00)	0.045
MMSE	24.5 ± 4.2 (26.0)	22.7 ± 4.8 (23.0)	0.050	23.4 ± 4.8 (24.0)	24.1 ± 3.7 (25.0)	0.803
**Aspects related to pd**						
Disease duration (years)	8.0 ± 4.1 (8.0)	12.0 ± 7.0 (11.0)	0.003	8.8 ± 4.9 (8.0)	13.5 ± 7.7 (13.0)	0.006
HY			0.025			0.225
0‒2	17 (31 %)	6 (13 %)		20 (25 %)	3 (13 %)	
2.5‒3	38 (69 %)	42 (87 %)		60 (75 %)	20 (87 %)	
SE ADL			0.030			0.202
< 80 %	5 (9.1 %)	12 (25 %)		11 (14 %)	6 (26 %)	
> 80 %	50 (91 %)	36 (75 %)		69 (86 %)	17 (74 %)	
Levodopa Dosage (mg/day)	741 ± 29 (800)	786 ± 361 (800)	0.533	730 ± 299 (800)	877 ± 401 (950)	0.092
Dysautonomia	36 (65 %)	42 (88 %)	0.009	59 (74 %)	19 (83 %)	0.382
Visual hallucination			0.039			0.239
Yes	7 (13 %)	14 (29 %)		14 (18 %)	7 (30 %)	
No	48 (87 %)	34 (71 %)		66 (83 %)	16 (70 %)	
UPDRS III	37 ± 12 (34)	42 ± 14 (43)	0.032	44 ± 14 (44)	46 ± 14 (48)	0.529
PIGD	4.6 ± 2.5 (4.00)	6.2 ± 2.2 (6.00)	<0.001	5.26 ± 2.6 (5.00)	5.6 ± 2.3 (6.0)	0.398
LLB	6.2 ± 3.0 (6.0)	7.2 ± 3.5 (7.0)	0.112	6.4 ± 3.2 (6.0)	7.7 ± 3.5 (7.0)	0.122
**Aspects related to sarcopenia**						
SARC-F	2.87 ± 2.30 (2.00)	5.38 ± 2.54 (5.00)	<0.001	3.66 ± 2.66 (3.00)	5.35 ± 2.52 (5.00)	0.007
Grip strength (kg)	31 ± 11 (32)	27 ± 10 (25)	0.021	29 ± 10 (28)	28 ± 11 (27)	0.598
DEXA (ALM/height2 (kg/m^2^))	7.72 ± 1.28 (7.90)	7.10 ± 0.90 (7.10)	0.003	7.4 ± 1.1 (7.3)	7.5 ± 1.1 (7.5)	0.878
Walking speed	0.98 ± 0.20 (0.97)	0.79 ± 0.25 (0.80)	<0.001	0.90 ± 0.24 (0.90)	0.82 ± 0.21 (0.80)	0.024

1 Median (IQR) or Frequency (%) 2 Chi-Square test of independence; Wilcoxon rank sum test; Test Fisher's exact ^a^ At least 30-minutes 3 × *a* week.

COPD, Chronic Obstructive Pulmonary Disease; SARC-F, Strength, Assistance with walking, Rising from a chair, Climbing stairs, and Falls; GDS, Geriatric Depression Scale; MMSE, Mini-Mental State Examination; HY, Hoehn & Yahr; SE, Schwab and England; UPDRS-III, Unified Parkinson’s Disease Rating Scale part-III; PIGD, Postural Instability Gait Difficulty; LLB score, Lower Limb Bradykinesia score; SPPB, Short Physical Performance Battery; BMI, Body Mass Index; ALM/Ht2 kg/m^2^, Appendicular Lean Mass per squared Height.

Logistic regression was performed to assess variables independently associated with the outcome of falls and recurrent falls, considering those that exhibited statistically significant associations in the bivariate analysis. Although there was no significant difference in distribution between the groups, gender, age and HY were included in the logistic regression analysis as it was deemed important to control for these variables in the analysis of the others. In this regard, longer disease duration, history of falls in the last 6-months, and dysautonomia were independent predictors of falls (1 or more). Regarding recurrent falls, higher scores on the SARC-F, type-2 Diabetes and longer disease duration remained in the final model, as shown in Table 2. The variables with high correlation were removed from the model.

**Table 2:** Multivariate logistic regression of recurrent falls adjusted for age, sex and Hoehn & Yahr.

Variables	HR	95 % CI	p	VIF
Recurrent falls				
Depression	0.90	0.12, 5.03	0.905	1.2
Total SARC-F	1.38	1.04, 1.88	0.034	2.0
History of falls last 6-months	1.34	0.29, 4.90	0.773	1.5
Disease duration	1.17	1.06, 1.34	0.005	1.3
Gait speed	2.66	0.65, 11.8	0.151	1.9
Diabetes	9.49	1.53, 82.3	0.021	1.5
Dysautonomia	0.00		0.990	1.0
Falls (at least once)				
Depression	0.41	0.05, 2.93	0.386	1.6
Schwab & England				2.3
< 80	‒	‒		
> −80	1.04	0.11, 9.72	0.974	
Total SARC-F	1.34	0.93, 1.97	0.116	2.7
History of falls in the last 6-months	4.82	1.17, 24.2	0.038	1.5
Disease duration	1.15	1.02, 1.32	0.029	1.5
Gait speed	0.92	0.15, 4.95	0.924	2.4
UPDRS-III	0.93	0.86, 1.01	0.090	5.4
Alcohol intake				1.5
Yes	‒	‒		
No	3.09	0.36, 35.7	0.321	
Use of walking aid				1.2
Yes	‒	‒		
No	0.46	0.09, 2.05	0.317	
At least 30-minutes of exercise 3× week				1.6
Yes	‒	‒		
No	0.90	0.21, 3.76	0.879	
Visual Hallucinations				2.1
Yes	‒	‒		
No	0.66	0.07, 5.03	0.694	
Dysautonomia				1.6
No	‒	‒		
Yes	10.0	1.91, 75.9	0.012	
Number of medicines	1.13	0.79, 1.61	0.491	1.5
SPPB	0.95	0.66, 1.36	0.785	2.4
MMSE	0.93	0.77, 1.12	0.436	2.6
GDS	1.09	0.87, 1.36	0.460	1.7
Antihypertensives	0.38	0.08, 1.77	0.224	1.6
Benzodiazepines	1.22	0.06, 71.7	0.905	1.4

HR, Hazard Ratio; CI, Confidence Interval; VIF, Variance Inflation Factor; SARC-F, Strength, Assistance with walking, Rising from a chair, Climbing stairs, and Falls; UPDRS-III, Unified Parkinson’s Disease Rating Scale part-III; SPPB, Short Physical Performance Battery; MMSE, Mini-Mental State Examination; GDS, Geriatric Depression Scale.

**Fig. 2:**
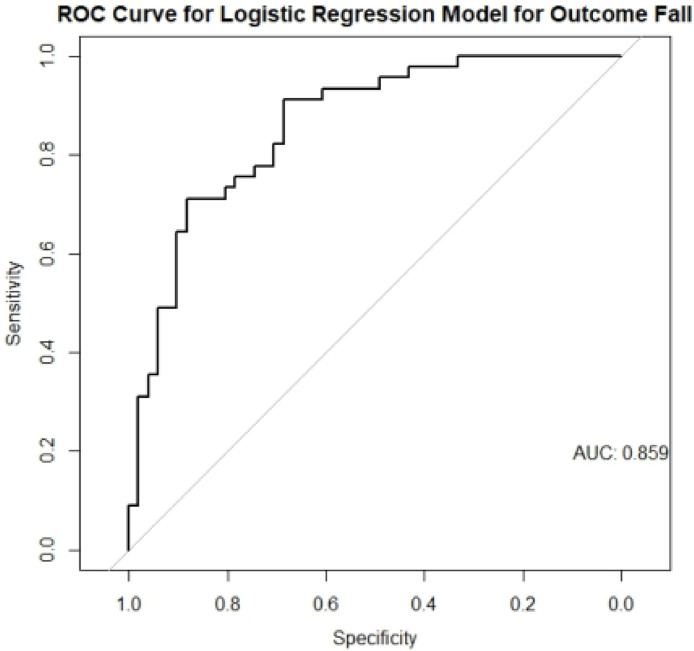
A Receiver Operating Characteristic (ROC) analysis for SARC-F score and disease duration as predictors of recurrent falls.

A Receiver Operating Characteristic (ROC) analysis was performed for SARC-F score and disease duration as predictors of recurrent falls, and the respective curve is shown in Fig. 2. This model showed a good accuracy with an area under the curve of 0.843.

The authors evaluated the accuracy of each question of the SARC-F (Table 3). The item of the frequency of falls in the last year was the highest accuracy one, with cut-off points 1 and 2 with 61.17 % and 78.64 %, respectively. The authors also calculated the accuracy of the total SARC-F (Table 3) in all possible cut-off points above 4 (cut-off point used as positive for sarcopenia). The accuracy increases progressively according to the cut-off point, starting with 58.06 % for the cut-off point of 4 and ending with 78.64 % with the maximum score of 10.

**Table 3:** Accuracy of each question and total SARC-F to predict recurrent falls in twelve months.

			Cutoff Score						
Question			≥1				2		
1st Handgrip strength			50.49 %				68.93 %		
2nd Mobility			54.37 %				73.79 %		
3rd Chair-stand			42.72 %				71.84 %		
4th Climb stair			47.57 %				62.14 %		
5th Falls last year			61.17 %				78.64 %		

Cutoff Score									
SARC-F	4	5		6	7	8		9	10
Accuracy	58.06 %	63.11 %		67.96 %	70.87 %	72.82 %		76.7 %	78.64 %

SARC-F: strength, assistance 6- with walking, rising from a chair, climbing stairs, and falls.

The authors analyzed the correlation among sarcopenia screening test (SARC-F), grip strength, and SPPB with UPDRS-III and its subtopics representing LLB and PIGD pattern (Table 4). Statistically significant correlations were observed between SARC-F and disease severity scale (UPDRS-III) and between SARC-F and LLB and PIGD pattern, with correlation coefficients of 0.479, 0.469, and 0.518, respectively.

**Table 4:** Spearman’s correlation between screening tests of sarcopenia and UPDRS.

Parameter 1	Parameter 2	rho	CI_low	CI_high	p
LLB score	Total SARC-F	0.469	0.301	0.608	**0.000**
LLB	Grip strength	−0.213	−0.394	−0.015	**0.030**
LLB	SPPB	−0.315	−0.483	−0.124	**0.001**
UPDRS Part III	Total SARC-F	0.479	0.309	0.619	**0.000**
UPDRS Part III	Grip strength	−0.207	−0.393	−0.005	**0.039**
UPDRS Part III	SPPB	−0.395	−0.552	−0.211	**0.000**
PIGD	Total SARC-F	0.518	0.359	0.648	**0.000**
PIGD	Grip strength	−0.326	−0.493	−0.137	**0.001**
PIGD	SPPB	−0.546	−0671	−0.390	**0.000**
Total SARC-F	Grip strength	−0.327	−0.493	−0.138	**0.001**
Total SARC-F	SPPB	−0.558	−0.681	−0.405	**0.000**
Grip strength	SPPB	0.362	0.174	0.525	**0.000**

LLB score, Lower Limb Bradykinesia score; UPDRS-III, Unified Parkinson’s Disease Rating Scale part-III; PIGD, Postural Instability Gait Difficulty; SPPB, Short Physical Performance Battery.

Table 5 describes a summary of the circumstances of the falls reported on the calls. From the total of 159 falls, 89 (56 %) occurred in women, most of them (*n* = 139; 87 %) did not require medical care, only 2 (1 %) resulted in hospitalization, and one patient (0.6 %) had a hip fracture. Most of the falls happened during the morning time (*n* = 123; 77 %) and in the interior part of their house (*n* = 115; 72 %). Also, 69 (43 %) of the falls were due to imbalance.

**Table 5:** Characteristics and circumstances of falls.

Variables	Falls (*n* = 159)
**Sex**	
Male	70 (44 %)
Female	89 (56 %)
**Sought medical care**	
No	139 (87 %)
Yes	19 (13 %)
**Place of medical care**	
Emergency	14 (9 %)
Ambulatory	4 (3 %)
**Hospitalized**	
No	17 (11 %)
Yes	2 (1 %)
**Injury**	
No	105 (66 %)
Laceration	43 (27 %)
Dislocation	6 (3,7 %)
Other fracture	3 (2 %)
Hip	1 (0,6 %)
Head trauma	1 (0,6 %)
**Time**	
Morning	123 (77 %)
Night	27 (17 %)
Evening	2 (1,2 %)
**Location of fall**	
Interior part of the house	115 (72 %)
Exterior part of the house	25 (16 %)
Outdoors	14 (9 %)
**Distribution of falls in relation to environment**	
Forward	58 (36 %)
Backward	53 (33 %)
Sideways	35 (22 %)
Kneeling	4 (2,5 %)
Sitting	3 (2 %)
**Circumstances**	
Tripping	22 (14 %)
Slipping	22 (14 %)
Distraction, uneven terrain	1 (0,6 %)
Imbalance	69 (43 %)
Lower limb weakness	23 (14 %)
Vertigo	17 (11 %)

**Fig. 3:**
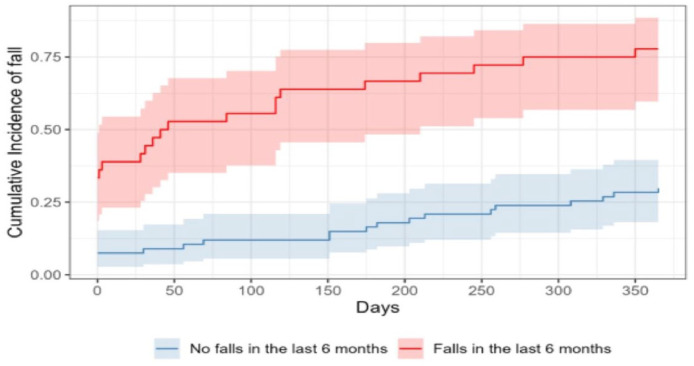
Cumulative incidence of falls of patients with and without history of previous falls in the last 6-months.

Fig. 3 shows the cumulative incidence of falls of patients with and without history of previous falls in the last 6-months. The patients with a history off previous falls in the previous 6-months showed a significantly higher cumulative incidence over the course of 12-months (Log-rank test, *p* < 0.001). By the time the study reached its half-year mark, the incidence in the group without a history of falls was 16.42 %, while the group with a previous history was 66.7 %. The cumulative incidence at the end of the 12-month period was 29.85 % in the group with no history of previous falls and 77.8 % in the group with previous falls.

## Discussion

The incidence of single and repeated falls is comparable to that reported by others^11^: 46.6 % of our sample fell at least once in a year, and 22.3 % fell more than once. Longer disease duration was a predictor of recurrent falls and falls (at least once) in a period of 12-months. Previous falls in the last 6 months and dysautonomia also predicted falls. The SARC-F and type-2 diabetes predicted recurrent falls in the logistic regression.

Patients with longer disease duration have greater disability, higher risk of falling, and unfavorable outcomes such as fracture and hospitalization.^12^ The history of previous falls stands out as the most robust risk factor for the occurrence of future falls.^11^^,^^13^, ^14^, ^15^ Other predictors include cognitive impairment, with a 1.2- to 1.5-fold increased risk for each point lost on the MMSE^11^ and FOG.^11^^,^^13^^,^^15^ The use of certain drugs by patients was also related to a higher risk of falls, such as levodopa equivalent doses greater than 700 mg/day,^13^ the use of COMT inhibitors,^13^ amantadine,^13^ benzodiazepines,^13^ atypical antipsychotics,^13^ and antidepressants.^13^ In addition, factors such higher scores on the UPDRS,^11^^,^^13^ on the questionnaires which assess quality of life like Parkinson's Disease Questionnaire 39 and Parkinson's Disease Questionnaire 8,^11^ GDS-15,^11^ as well as HY greater than 2.5,^11^^,^^12^ SE less than or equal to 80 %,^13^ presence of dyskinesias,^11^^,^^13^ dysautonomia,^13^ history of “off”,^11^^,^^13^ were also associated as predictors of falls in PD.

Previous studies have demonstrated an association of SARC-F and falls in community-dwelling older adults^16^ and as an independent predictor of falls in post-menopausal women.^17^ A retrospective cohort study involving 9927 patients aged 65 and older in Japan found that in-hospital falls were substantially more common in patients with a SARC-F score of ≥ 2 than in those with a score of < 2 (3.7 % vs. 0.7 %, *p* < 0.001). Furthermore, the hazard ratio for falls was considerably greater in those with a SARC-F score of ≥ 2 (2.11 [1.37‒3.15], *p* < 0.001). These findings suggest that the SARC-F can be used to predict falls in older persons who are hospitalized.^18^

The question in the SARC-F's regarding falls of “How many times have you fallen in the past year?” is scored from 0 to 2, with 0 indicating no falls and 2 indicating four or more falls. When the history of falls question scores 2, it indicates a higher risk of falling and may indicate the need for additional examination and action to prevent falls. This is the most important SARC-F question for predicting falls and recurrent falls.

Having four or more previous falls was found to be the strongest predictor factor in a study by Wapp et al. (2022),^12^ suggesting that those with a history of many falls are more likely to fall multiple times again. A single fall may happen at random, but those who fall frequently are probably dealing with long-term problems that make it difficult for them to avoid falling. Other studies have emphasized the history of falls in fall prediction.^19^^,^^20^

In a prospective longitudinal study^20^ conducted in Hong Kong, 4000 Chinese individuals 65 years of age or older who lived in the community (2000 of them were men) discovered that the SARC-F evaluation instrument strongly predicted five poor outcomes for women in 4 years (worsened physical limitations, recurrent falls, living in nursing home, days of hospital stay ≥ 10, Short Form Health Survey questionnaire (SF-12) mental component summary decline ≥ 5) and six adverse outcomes for men in four years, with the exception of mortality, which was reduced in ten years (worsened physical limitations, recurrent falls, days of hospital stay ≥ 10, SF-12 mental component summary decline ≥5 and mortality in ten years). The prediction for all outcomes in both genders was (marginally) significantly improved by including both grip strength and gait speed in the analysis compared to SARC-F alone, except for recurrent falls. They also found that, in addition to SARC-F, adding grip strength and chair-stand produced a (marginally) significant extra prediction value for all outcomes except for recurrent falls in both genders and serious osteoporotic fractures in women. Additionally, they observed that regardless of the correction parameters, measuring muscle mass with DEXA in addition to SARC-F, muscle strength, and function does not improve the prediction of unfavorable outcomes.

The occurrence of sarcopenia among individuals with PD may result in an increased vulnerability to falling and subsequent disability.^21^ A notable association was observed between higher scores obtained from the SARC-F questionnaire, which is employed to evaluate sarcopenia, and the presence of disabilities in PD patients.^22^

Nevertheless, a few studies have shown that the SARC-F has poor specificity for sarcopenia diagnosis in people with PD. Notably, Parkinsonian symptoms such as stiffness, bradykinesia, postural instability, and orthostatic hypotension may be the reason for difficulties performing the activities evaluated in the SARC-F. The SARC-F is not well-established in this setting for this population.^22^

The authors found a correlation between motor symptoms assessed through the UPDRS-III and its subdivisions (LLB and PIGD) with the SARC-F score. Other studies have previously shown an association between muscle function and the severity of PD.^9^^,^^23^ There is a decrease in the person's strength when the motor symptoms, as assessed by the UPDRS-III, worsen.

Diabetes remained an independent predictor of recurrent falls in the multivariate analysis. Diabetes and PD exacerbate each other's effects on motor and neurocognitive functions.^23^ A recent meta-analysis found that diabetes was linked to an increased risk and accelerated progression of PD.^24^

Diabetes is associated with several complications, functional decline, and a higher risk of frailty.^25^ Older adults with T2DM have an increased risk of falls and recurrent falls^26^ probably due to the presence of neuropathy,^27^ retinopathy,^28^ orthostatic hypotension,^29^ hypoglycemia.^29^ The diabetic polyneuropathy causes numbness, loss of sensibility, and postural stability, and neuropathic pain.^30^

The authors looked specifically at the following clinical conditions while investigating dysautonomia: postural hypotension, intestinal constipation, and urge incontinence. Dysautonomia is an essential factor in the development of Orthostatic Hypotension (OH) that causes cerebral hypoperfusion, which can either directly or indirectly increase the risk of falling.^31^ Syncopal events were excluded from the operational definition of a fall in this study, but hypoperfusion symptoms such as dizziness and loss of balance are contributors to falls.

Neikrug and colleagues suggested that PD patients with RBD have a higher prevalence of constipation (72 % vs. 50 %), as well as more severe alpha-synucleinopathy in the brain, limited cognitive dysfunction, orthostatic hypotension, and cardiac sympathetic denervation.^32^ Moreover, studies have shown that the presence of constipation correlates with a decline in cognitive function, which is another risk factor for falls in PD patients.^33^

Urinary Incontinence (UI) is a common non-motor symptom in PD that significantly impacts the quality of life and is associated with an increased risk of falls.^34^ This urgency often leads to hurried movements, which, coupled with the motor deficits of PD such as bradykinesia, postural instability, and freezing of gait, dramatically elevate the risk of falls.^35^

To the best of our knowledge, this is the first study that evaluated the SARC-F as a predictor of falls in PD, but this study has certain limitations. First, there may have been an underdiagnosis of falls since many patients were uninterested in sharing information, forgot to fill out the given questionnaire about the circumstances of the falls, and required reminders to recall the description of the fall during the call. The monthly check-ins also meant that fall prevention received more attention.

The authors acknowledge the possibility of overestimation of the amount of lean mass due to edema or fat. The authors tried to reduce this bias through a medical consultation that included a physical examination to check for edema, as well as by excluding clinical conditions that cause edema, such as severe heart failure, severe renal dysfunction, neoplasms, morbid obesity, and acute conditions.

More prospective longitudinal research on falls is required to reduce this information bias, with the incidence of falls being recorded using technology. The lack of a control group, which would have been crucial for assessing how sarcopenia variables would have behaved in a group matched by age and sex but not by PD, was another limitation. Another bias of the present study was the subjectivity of the information provided by the patient regarding physical activity, which is an important protective factor for muscle strength and quality.

## Conclusions

Higher SARC-F, longer disease duration, and type-2 diabetes were independent predictors of recurrent falls. History of falls in the last 6-months, longer disease duration, and dysautonomia were independent predictors of falls (at least once) in a prospective study of 12-months. Confirmed sarcopenia didn’t predict falls or recurrent falls in the present cohort. More robust prospective studies with a control group and specific tools to evaluate sarcopenia in PD are required. As motor function deteriorates, as measured by the UPDRS, there is a reduction in muscle function, as found in other studies that also used dynamometry as a parameter for assessing muscle strength.

## Ethical publication statement

The authors confirm that we have read the Journal's position on issues involved in ethical publication and affirm that this work is consistent with those guidelines. The authors confirm that the Research Ethics Committee of Hospital Universitário Walter Cantidio, Fortaleza, Ceará, Brazil, approved this study (register n° 91,075,318.1.0000.5045, June 1, 2018). The authors confirm that the participants provided written informed consent before the data were collected.

## Data availability

The authors confirm that the data supporting the findings of this study are available within the article and its Supplementary Materials. Raw data were collected and managed using the REDCap electronic data collection and management tool hosted at the Clinical Research Unit of the Hospital Universitário Walter Cantídio. Derived data supporting the findings of this study are available from the corresponding author, DPL on request.

## Authors’ contributions

Danielle Pessoa Lima: Conceptualization; methodology; data curation; investigation, writing-original draft preparation; writing-review & editing; project administration.

Vlademir Carneiro Gomes: Data curation; Investigation; writing-original draft preparation; writing-review & editing.

João Rafael Gomes de Luna: Data curation; investigation; writing-original draft preparation; writing-review & editing.

Lucas Tadeu Rocha Santos: Data curation; investigation; writing-original draft preparation; writing-review & editing.

Samuel Brito de Almeida: Investigation; writing-original draft preparation; writing-review & editing.

Antonio Brazil Viana-Júnior: Formal analysis.

Carlos Eduardo Urbano da Silva: Investigation; writing-original draft preparation; writing-review & editing.

Thais de Menezes Dantas: Investigation.

Carla Marineli Saraiva do Amaral: Investigation.

Arnaldo Aires Peixoto Júnior: Writing-original draft preparation; writing-review & editing.

Jarbas de Sá Roriz-Filho: Writing-original draft preparation; writing-review & editing.

Renan Magalhaes Montenegro-Júnior: Resources.

Pedro Braga-Neto: Supervision; resources; project administration.

## Funding sources

There was no financial support for the execution of this work.

Financial disclosures for the last 12-months: The authors declare that there are no additional disclosures to report.

## Declaration of competing interest

The authors declare no conflicts of interest.

## Appendix Supplementary materials

Image, application 1
