# Supplementary material for: Assessment of sarcopenia tools as predictors of falls in patients with mild to moderate Parkinson's Disease: A cohort study
Source: Clinics (Sao Paulo). 2025 Sep 18;80:100776. doi: 10.1016/j.clinsp.2025.100776 (PMC12477909; doi:10.1016/j.clinsp.2025.100776)
Supplement: Supplementary file 1 [file mmc1.docx]

**CLINICS-D-24-01239**

**Supplementary Material**

**Supplementary Table** Bivariate analysis of Clinical and demographical variables with falls and recurrent falls.

|  | **Fall** | | | | **Recurrent fall** | | | |
| --- | --- | --- | --- | --- | --- | --- | --- | --- |
| **Variables** | **N** | **0 fall**  **(n = 55)^a^** | **≥ 1 fall**  **(n = 48)^a^** | **p-value^b^** | **n** | **0‒1 fall**  **(n = 80)^a^** | **≥ 2 fall**  **(n = 23)^a^** | **p-value^b^** |
| ***CLINICAL ASPECTS*** |  |  |  |  |  |  |  |  |
| **Gender** | 103 |  |  | 0.348 | 103 |  |  | 0.458 |
| Female |  | 18 (33%) | 20 (42%) |  |  | 28 (35%) | 10 (43%) |  |
| Male |  | 37 (67%) | 28 (58%) |  |  | 52 (65%) | 13 (57%) |  |
| **Age** | 103 | 66 ± 10 (63) | 66 ± 11 (69) | 0.997 | 103 | 66 ± 11 (69) | 65 ± 10 (65) | 0.716 |
| **Hypertension** | 103 | 28 (51%) | 18 (38%) | 0.172 | 103 | 37 (46%) | 9(39%) | 0.545 |
| **Type-2 Diabetes** | 103 | 4 (7.3%) | 6 (13%) | 0.508 | 103 | 5 (6.3%) | 5 (22%) | **0.042** |
| **Dyslipidemia** | 103 | 8 (15%) | 6 (13%) | 0.763 | 103 | 12 (15%) | 2 (8.7%) | 0.730 |
| **Dementia (Azheimer, Vascular)** | 103 | 0 (0%) | 0 (0%) |  | 103 | 0 (0%) | 0 (0%) |  |
| **COPD** | 103 | 0 (0%) | 1 (2.1%) | 0.468 | 103 | 0 (0%) | 1 (4.3%) | 0.223 |
| **Uncorrected hearing deficit** | 103 | 3 (5.5%) | 6 (13%) | 0.298 | 103 | 6 (7.5%) | 3 (13%) | 0.414 |
| **Uncorrected visual deficit** | 103 | 5 (9.1%) | 1 (2.1%) | 0.212 | 103 | 5 (6.3%) | 1 (4.3%) | >0.999 |
| **Alcoholism** | 103 |  |  | **0.009** | 103 |  |  | 0.184 |
| Yes |  | 12 (22%) | 2 (4.2%) |  |  | 13 (16%) | 1 (4.3%) |  |
| No |  | 43 (78%) | 46 (96%) |  |  | 67 (84%) | 22 (96%) |  |
| **Smoking** | 103 |  |  | >0.999 | 103 |  |  | 0.398 |
| Yes |  | 1 (1.8%) | 1 (2.1%) |  |  | 1 (1.3%) | 1 (4.3%) |  |
| No |  | 54 (98%) | 47 (98%) |  |  | 79 (99%) | 22 (96%) |  |
| **Use of walking aid** | 103 |  |  | **0.006** | 103 |  |  | 0.088 |
| Yes |  | 6 (11%) | 16 (33%) |  |  | 14 (18%) | 8 (35%) |  |
| No |  | 49 (89%) | 32 (67%) |  |  | 66 (83%) | 15 (65%) |  |
| **Physical therapy** | 103 |  |  | 0.298 | 103 |  |  | 0.414 |
| Yes |  | 3 (5.5%) | 6 (13%) |  |  | 6 (7.5%) | 3 (13%) |  |
| No |  | 52 (95%) | 42 (88%) |  |  | 74 (93%) | 20 (87%) |  |
| **At least 30 minutes of exercise 3× week *** | 103 |  |  | **0.006** | 103 |  |  | 0.814 |
| Yes |  | 32 (58%) | 15 (31%) |  |  | 37 (46%) | 10 (43%) |  |
| No |  | 23 (42%) | 33 (69%) |  |  | 43 (54%) | 13 (57%) |  |
| **Depression** | 103 |  |  | **0.009** | 103 |  |  | **0.025** |
| Yes |  | 10 (18%) | 20 (42%) |  |  | 19 (24%) | 11 (48%) |  |
| No |  | 45 (82%) | 28 (58%) |  |  | 61 (76%) | 12 (52%) |  |
| **Number of medicines** | 103 | 4.83 ± 2.13 (5.00) | 5.75 ± 2.24 (5.50) | **0.039** | 102 | 5.10 ± 2.11 (5.00) | 5.74 ± 2.56 (5.00) | 0.372 |
| **Antidepressives** | 103 | 17 (31%) | 24 (50%) | **0.048** | 103 | 28 (35%) | 13 (57%) | 0.063 |
| **Antihypertensives** | 103 | 23 (42%) | 11 (23%) | **0.042** | 103 | 30 (38%) | 4 (17%) | 0.071 |
| **Benzodiazepines** | 103 | 2 (3.6%) | 9 (19%) | **0.013** | 103 | 6 (7.5%) | 5 (22%) | 0.065 |
| **Typical antipsychotics** | 103 | 0 (0%) | 0 (0%) |  | 103 | 0 (0%) | 0 (0%) |  |
| **Atypical antipsychotics** | 103 | 2 (3.6%) | 2 (4.2%) | >0.999 | 103 | 3 (3.8%) | 1 (4.3%) | >0.999 |
| **Anticonvulsants** | 103 | 2 (3.6%) | 5 (10%) | 0.247 | 103 | 5 (6.3%) | 2 (8.7%) | 0.651 |
| **Anticholinesterase** | 103 | 0 (0%) | 5 (10%) | **0.020** | 103 | 3 (3.8%) | 2 (8.7%) | 0.310 |
| **GDS** | 101 | 4.11 ± 2.95 (4.00) | 6.00 ± 3.52 (5.50) | **0.005** | 101 | 4.60 ± 3.11 (4.00) | 6.39 ± 3.83 (6.00) | **0.045** |
| **MMSE** | 99 | 24.5 ± 4.2 (26.0) | 22.7 ± 4.8 (23.0) | **0.050** | 99 | 23.4 ± 4.8 (24.0) | 24.1 ± 3.7 (25.0) | 0.803 |
| ***ASPECTS RELATED TO PD*** |  |  |  |  |  |  |  |  |
| **Disease duration** | 103 | 8.0 ± 4.1 (8.0) | 12.0 ± 7.0 (11.0) | **0.003** | 103 | 8.8 ± 4.9 (8.0) | 13.5 ± 7.7 (13.0) | **0.006** |
| **HY** | 103 |  |  | **0.025** | 103 |  |  | 0.225 |
| 0‒2 |  | 17 (31%) | 6 (13%) |  |  | 20 (25%) | 3 (13) |  |
| 2.5‒3 |  | 38 (69%) | 42 (87%) |  |  | 60 (75%) | 20 (87) |  |
| **SE ADL** | 103 |  |  | **0.030** | 103 |  |  | 0.202 |
| < 80 |  | 5 (9.1%) | 12 (25%) |  |  | 11 (14%) | 6 (26%) |  |
| > -80 |  | 50 (91%) | 36 (75%) |  |  | 69 (86%) | 17 (74%) |  |
| **Autonomic dysfunction** | 103 |  |  | **0.009** | 103 |  |  | 0.382 |
| Yes |  | 36 (65%) | 42 (88%) |  |  | 59 (74%) | 19 (83%) |  |
| No |  | 19 (35%) | 6 (13%) |  |  | 21 (26%) | 4 (17%) |  |
| **Visual hallucination?** | 103 |  |  | **0.039** | 103 |  |  | 0.239 |
| Yes |  | 7 (13%) | 14 (29%) |  |  | 14 (18%) | 7 (30%) |  |
| No |  | 48 (87%) | 34 (71%) |  |  | 66 (83%) | 16 (70%) |  |
| **Freezing of gait complaint** | 102 |  |  | 0.200 | 102 |  |  | 0.532 |
| Yes |  | 20 (36%) | 23 (49%) |  |  | 32 (41%) | 11 (48%) |  |
| No |  | 35 (64%) | 24 (51%) |  |  | 47 (59%) | 12 (52%) |  |
| **Dyskinesia** | 103 |  |  | 0.485 | 103 |  |  | 0.772 |
| Yes |  | 26 (47%) | 26 (54%) |  |  | 41 (51%) | 11 (48%) |  |
| No |  | 29 (53%) | 22 (46%) |  |  | 39 (49%) | 12 (52%) |  |
| **Levodopa dosage** | 100 | 741 ± 298 (800) | 786 ± 361 (800) | 0.533 | 100 | 730 ± 299 (800) | 877 ± 401 (950) | 0.092 |
| **Pramipexole dosage** | 52 | 2.17 ± 1.46 (1.50) | 2.33 ± 1.08 (2.00) | 0.462 | 52 | 2.08 ± 1.29 (1.50) | 2.73 ± 1.10 (3.00) | 0.090 |
| **Amantadine dosage** | 33 | 250 ± 74 (200) | 322 ± 67 (300) | 0.174 | 33 | 273 ± 96 (300) | 322 ± 67 (300) | 0.174 |
| **Entacapone dosage** | 32 | 967 ± 239 (1,000) | 940 ± 252 (1,000) | 0.823 | 33 | 927 ± 235 (1,000) | 1,000 ± 267 (1,000) | 0.408 |
| **UPDRS III** | 103 | 37 ± 12 (34) | 42 ± 14 (43) | **0.032** | 99 | 44 ± 14 (44) | 46 ± 14 (48) | 0.529 |
| **UPDRS III subscore – Stiffness** | 103 | 4.89 ± 2.26 (5.00) | 4.65 ± 2.20 (4.00) | 0.578 | 103 | 4.91±2.25 (5.00) | 4.30±2.10 (4.00) | 0.327 |
| **UPDRS III subscore – Arising from chair** | 103 | 1.51 ± 0.72 (1.00) | 1.81 ± 0.79 (2.00) | **0.024** | 103 | 1.63 ± 0.77 (2.00) | 1.74 ± 0.75 (2.00) | 0.410 |
| **UPDRS III subscore – Gait** | 103 | 2.40 ± 0.53 (2.00) | 2.69 ± 0.55 (3.00) | **0.013** | 103 | 2.50 ± 0.57 (2.00) | 2.65 ± 0.49 (3.00) | 0.206 |
| **UPDRS III subscore – Freezing of gait** | 103 | 1.18 ± 0.39 (1.00) | 1.31 ± 0.59 (1.00) | 0.332 | 103 | 1.21 ± 0.44 (1.00) | 1.35 ± 0.65 (1.00) | 0.446 |
| **UPDRS III subscore – Postural stability** | 103 | 2.18 ± 1.02 (2.00) | 2.75 ± 1.18 (2.50) | **0.013** | 103 | 2.36 ± 1.09 (2.00) | 2.74 ± 1.21 (2.00) | 0.191 |
| **UPDRS III subscore – Posture** | 103 | 2.51 ± 1.10 (2.00) | 2.96 ± 1.01 (3.00) | **0.033** | 103 | 2.78 ± 1.10 (3.00) | 2.52 ± 0.99 (2.00) | 0.322 |
| **UPDRS III subscore: Global spontaneity of Movement** | 103 | 2.43 ± 0.81 (2.00) | 2.89 ± 0.84 (3.00) | **0.005** | 103 | 2.59 ± 0.87 (2.00) | 2.83 ± 0.78 (3.00) | 0.193 |
| ***ASPECTS RELATED TO SARCOPENIA*** |  |  |  |  |  |  |  |  |
| **SARC-F** | 103 | 2.87 ± 2.30 (2.00) | 5.38 ± 2.54 (5.00) | **<0.001** | 103 | 3.66 ± 2.66 (3.00) | 5.35 ± 2.52 (5.00) | **0.007** |
| **Grip strength** | 103 | 31 ± 11(32) | 27 ± 10 (28) | 0.081 | 103 | 30 ± 11 (30) | 28 ± 9 (29) | 0.521 |
| **SPPB score** | 101 | 9.72 ± 2.33 (10.00) | 7.79 ± 2.70 (8.00) | **<0.001** | 101 | 8.91 ± 2.80 (9.00) | 8.52 ± 2.27 (9.00) | 0.351 |
| **Confirmed sarcopenia** | 100 | 8 (15%) | 4 (8.9%) | 0.387 | 100 | 10 (13%) | 2 (9.5%) | >0.999 |
| **Grip strength** | 103 |  |  | 0.917 | 103 |  |  | 0.393 |
| 0- Altered |  | 11 (20%) | 10 (21%) |  |  | 18 (22%) | 1. (13%) |  |
| 1- Normal |  | 44 (80%) | 38 (79%) |  |  | 62 (78%) | 20 (87%) |  |
| **SPPB** | 101 |  |  | **<0.001** | 101 |  |  | 0.205 |
| 2- ≤ 8 |  | 11 (20%) | 26 (55%) |  |  | 26 (33%) | 1. 48%) |  |
| 3- > 8 |  | 43 (80%) | 21 (45%) |  |  | 52 (67%) | 12 (52%) |  |
| **Balance (section 1 of SPPB)** | 101 |  |  | **0.002** | 101 |  |  | 0.149 |
| 0 - Unable |  | 1 (1.9%) | 2 (4.3%) |  |  | 3 (3.8%) | 0 (0%) |  |
| 1- Able only to stand feet together side-by-side for 10 seconds |  | 2 (3.7%) | 8 (17%) |  |  | 6 (7.7%) | 4 (17%) |  |
| 2- Score 1 plus able to stand heel of one foot against side of big toe of the other foot for 10 seconds |  | 3 (5.6%) | 11 (23%) |  |  | 9 (12%) | 5 (22%) |  |
| 3- Score 2 plus able to stand feet aligned heel to toe for 3‒9.99 sec |  | 4 (7.4%) | 4 (8.5%) |  |  | 5 (6.4%) | 3 (13%) |  |
| 4- Score 2 plus able to stand feet aligned heel to toe for 10 sec |  | 44 (81%) | 22 (47%) |  |  | 55 (71%) | 11 (48%) |  |
| **Gait speed test (section 2 of SPPB)** |  |  |  |  |  |  |  |  |
| Time required to walk 4 meters | 101 |  |  | **<0.001** | 101 |  |  | >0.999 |
| 0- Unable |  | 0 (0%) | 2 (4.3%) |  |  | 2 (2.6%) | 0 (0%) |  |
| 1- > 8.70 sec |  | 2 (3.7%) | 1 (2.1%) |  |  | 3 (3.8%) | 0 (0%) |  |
| 2- 6.21 to 8.70 sec |  | 4 (7.4%) | 4 (8.5%) |  |  | 6 (7.7%) | 2 (8.7%) |  |
| 3- 4.82 to 6.20 sec |  | 3 (5.6%) | 5 (11%) |  |  | 6 (7.7%) | 2 (8.7%) |  |
| 4- < 4.82sec |  | 45 (83%) | 35 (74%) |  |  | 61 (78%) | 19 (83%) |  |
| **Chair stand test (section 3 of SPPB)** |  |  |  |  |  |  |  |  |
| Time required to perform 5 rises from a chair | 101 |  |  | **0.045** | 101 |  |  | 0.470 |
| 0- 60 sec or unable |  | 3 (5.6%) | 10 (21%) |  |  | 11 (14%) | 2 (8.7%) |  |
| 1- > 16.7 sec |  | 18 (33%) | 20 (43%) |  |  | 28 (36%) | 10 (43%) |  |
| 2- 13.70 to 16.69 sec |  | 5 (9.3%) | 4 (8.5%) |  |  | 5 (6.4%) | 4 (17%) |  |
| 3- 11.20 to 13.69 sec |  | 11 (20%) | 7 (15%) |  |  | 15 (19%) | 3 (13%) |  |
| 4- 11.19 sec or less |  | 17 (31%) | 6 (13%) |  |  | 19 (24%) | 4 (17%) |  |
| **Gait speed (m/s)** | 100 | 1.38 ±0.54 (1.40) | 1.15 ±0.45 (1.07) | **0.024** | 100 | 1.30 ± 0.53 (1.29) | 1.22± 0.45 (1.12) | 0.502 |
| **BMI (Body Mass Index)** | 100 | 25.9 ± 4.6 (25.8) | 26.9 ±5.4 (26.4) | 0.299 | 100 | 26.3 ±5.2 (26.3) | 26.5 ± 4.5 (26.1) | 0.536 |
| **ALM/Ht2 kg/m^2^** | 102 | 7.34 ± 1.15 (7.13) | 7.33 ± 1.29 (7.44) | 0.909 | 102 | 7.30 ± 1.16 (7.33) | 7.46 ± 1.40 (7.39) | 0.795 |
| **Low appendicular lean mass** | 102 | 14 (26%) | 7 (15%) | 0.157 | 102 | 18 (23%) | 3 (13%) | 0.391 |
| **Body mass densitometry** | 101 |  |  | 0.182 | 101 |  |  | 0.522 |
| Osteoporosis |  | 12 (22%) | 7 (15%) |  |  | 16 (21%) | 3 (13%) |  |
| Osteopenia |  | 20 (37%) | 26 (55%) |  |  | 33 (42%) | 13 (57%) |  |
| Normal |  | 22 (41%) | 14 (30%) |  |  | 29 (37%) | 7 (30%) |  |
| **Average protein intake g/kg/day** | 102 | 1.24 ± 0.37 (1.21) | 1.15 ± 0.44 (1.11) | 0.230 | 102 | 1.19 ± 0.39 (1.13) | 1.21 ± 0.47 (1.29) | 0.707 |
| **Right Calf circumference (cm)** | 100 | 33.5 ± 3.7 (33.5) | 33.5 ± 3.7 (33.0) | 0.898 | 100 | 33.6 ± 3.5 (33.5) | 33.3 ± 4.3 (33.0) | 0.712 |
| **First question of SARC-F** | 103 |  |  | **0.052** | 103 |  |  | 0.179 |
| 0 |  | 28 (51%) | 17 (35%) |  |  | 37 (46%) | 8 (35%) |  |
| 1 |  | 17 (31%) | 12 (25%) |  |  | 24 (30%) | 5 (22%) |  |
| 2 |  | 10 (18%) | 19 (40%) |  |  | 19 (24%) | 10 (43%) |  |
| **Second question of SARC-F** | 103 |  |  | **0.017** | 103 |  |  | 0.623 |
| 0 |  | 37 (67%) | 20 (42%) |  |  | 45 (56%) | 12 (52%) |  |
| 1 |  | 15 (27%) | 19 (40%) |  |  | 27 (34%) | 7 (30%) |  |
| 2 |  | 3 (5.5%) | 9 (19%) |  |  | 8 (10%) | 4 (17%) |  |
| **Third question of SARC-F** | 103 |  |  | **<0.001** | 103 |  |  | 0.102 |
| 0 |  | 20 (36%) | 9 (19%) |  |  | 25 (31%) | 4 (17%) |  |
| 1 |  | 31 (56%) | 19 (40%) |  |  | 40 (50%) | 10 (43%) |  |
| 2 |  | 4 (7.3%) | 20 (42%) |  |  | 15 (19%) | 9 (39%) |  |
| **Fourth question of SARC-F** | 103 |  |  | **0.003** | 103 |  |  | 0.105 |
| 0 |  | 24 (44%) | 8 (17%) |  |  | 29 (36%) | 3 (13%) |  |
| 1 |  | 19 (35%) | 16 (33%) |  |  | 25 (31%) | 10 (43%) |  |
| 2 |  | 12 (22%) | 24 (50%) |  |  | 26 (33%) | 10 (43%) |  |
| **Number of falls last year** | 103 |  |  | **<0.001** | 103 |  |  | **0.003** |
| None |  | 39 (71%) | 13 (27%) |  |  | 46 (58%) | 6 (26%) |  |
| Some / 1‒3 falls |  | 14 (25%) | 22 (46%) |  |  | 27 (34%) | 9 (39%) |  |
| Many or can’t remember / 4 or more falls |  | 2 (3.6%) | 13 (27%) |  |  | 7 (8.8%) | 8 (35%) |  |
| **Number of falls in past 6 months** | 103 | 0.36 ± 0.95 (0.00) | 3.38 ± 10.39 (1.00) | **<0.001** | 103 | 0.79 ± 1.73 (0.00) | 5.17 ± 14.73 (2.00) | **<0.001** |

^a^ Median (IQR) or Frequency (%).

^b^ Teste Qui-Quadrado de independência; Teste de soma de postos de Wilcoxon; Teste exato de Fisher.

COPD, Chronic Obstructive Pulmonary Disease; SARC-F, Strength, Assistance with walking, Rising from a chair, Climbing stairs, and Falls; GDS, Geriatric Depression Scale; MMSE, Mini-Mental State Examination; UPDRS-III, Unified Parkinson’s Disease Rating Scale; PIGD, Postural Instability Gait Difficulty; LLB score, Lower Limb Bradykinesia score; SPPB, Short Physical Performance Battery.
